# Advances and challenges in cancer immunotherapy: mechanisms, clinical applications, and future directions

**DOI:** 10.3389/fphar.2025.1602529

**Published:** 2025-06-13

**Authors:** Neeharika Vatsavai, Sumeet Kaur Bhinder, Rahaman Shaik, Shaheen Mahira, Shruti Kapoor, Md Shadab Ali, Deepak Verma, Jay Singh, Sreelakshmi Badavenkatappa Gari, Prabhat Upadhyay, Yeva Meshkovska, Chandraiah Godugu, Sowjanya Thatikonda, Venkatesh Pooladanda

**Affiliations:** ^1^ Department of Pharmacy, Birla Institute of Technology and Science (BITS) Pilani, Hyderabad Campus, Hyderabad, Telangana, India; ^2^ Department of Pharmaceutical Sciences and Drug Research, Punjabi University, Patiala, India; ^3^ Department of Pharmacology, School of Pharmaceutical Education and Research, Jamia Hamdard, New Delhi, India; ^4^ Department of Obstetrics, Gynecology and Reproductive Sciences, University of Pittsburgh, Pittsburgh, PA, United States; ^5^ Molecular, Cellular and Developmental Biology, University of California Santa Cruz, Santa Cruz, CA, United States; ^6^ Department of Pulmonary, Critical Care, and Sleep Medicine, All India Institute of Medical Sciences, New Delhi, India; ^7^ Department of Oncology, School of Medicine, Sidney Kimmel Comprehensive Cancer Center (SKCCC), Johns Hopkins Medicine, Baltimore, MD, United States; ^8^ Laboratory Oncology Unit, Dr. Bhim Rao Ambedkar Institute Rotary Cancer Hospital (Dr BRA-IRCH), All India Institute of Medical Sciences, New Delhi, India; ^9^ Faculty of Pharmaceutical Sciences, Jawaharlal Nehru Technological University Anantapur (JNTUA), Anantapur, Andhra Pradesh, India; ^10^ Department of Obstetrics & Gynecology, Vincent Center for Reproductive Biology, Massachusetts General Hospital, Boston, MA, United States; ^11^ Obstetrics, Gynecology & Reproductive Biology, Harvard Medical School, Boston, MA, United States; ^12^ Department of Head & Neck-Endocrine Oncology, Moffitt Cancer Center, Tampa, FL, United States; ^13^ Department of Regulatory Toxicology, Biological Sciences, National Institute of Pharmaceutical Education and Research (NIPER), Hyderabad, Telangana, India

**Keywords:** cancer immunotherapy, regulatory T cells, immune checkpoints, PD-1, CTLA-4, tumor immune evasion

## Abstract

Cancer is a major threat to public health today, particularly due to the emergence of drug resistance and disease re-emergence post-traditional treatment. Regulatory T cells (Tregs) support cancer progression through their immunosuppressive mechanisms expressing co-inhibitory molecules like programmed cell death-1 (PD-1), cytotoxic T lymphocyte-associated antigen 4 (CTLA-4), T cell immunoglobin-3 (TIM-3), and T-cell immunoglobin and ITIM domain (TIGIT), that suppress T-cell activation and allow tumor cells to grow uncontrollably. Emerging cancer immunotherapeutic strategies targeting these checkpoints inhibit tumor-immune escape and impede cancer progression. This review highlights the mechanistic effects of these drugs and enumerates various critical combinatorial strategies that can be utilized for effective cancer treatment.

## 1 Introduction to cancer immunotherapy

Cancer represents a major challenge to public health worldwide, with an estimated 2 million new cases in 2025, and a predicted mortality of over 618,120 people in the United States (Cancer Statistics, 2025). The World Health Organization (WHO) in 2022 projected around 20 million new cases and 9.5 million deaths from cancer globally (Authour Ananymous, 2024). Indeed, 1 out of every 5 individuals in the world has or will get cancer, and 1 out of every 9 male and 1 out of every 12 female patients will die of cancer (Global Cancer burden growing, amidst mounting need for services, 2024). Given the impact of socioeconomic disparities in cancer mortality and the coverage variability of Universal Health Coverage (UHC), there is a rising urgency and necessity to discover newer, accessible treatments every year. The current 5-year survival rate is 53.5 million people, highlighting the importance of this disease and the significance of new treatment discovery. Nonetheless, most countries’ funding for cancer prevention and emerging research remains low.

By overshadowing traditional therapy and providing advantage of progression-free survival and successful cancer amelioration, immunotherapy has emerged as a prominent leader in cancer therapy, facilitating development of novel approaches and treatment strategies (Tan et al., 2020). Immunotherapy is characterized by artificial enhancement or immune function-inhibition to cure diseases (Zhao et al., 2023). With ongoing research and development, immunotherapy is rapidly evolving and showing promising clinical results. However, limited FDA-approved treatments such as Immune checkpoint inhibitors (ICIs), Chimeric antigen receptor (CAR)-modified T cells, Neoantigen vaccines, and combination therapies are available for clinical use but elicit adverse effects such as inflammation, endocrine disorders, and autoimmunity (Yin et al., 2023). A multitude of biomarkers correlating the immune system’s response to treatment have been identified (Kichloo et al., 2021).

Understanding the key mechanisms underlying immunotherapy-related response and various host-tumor interactions is key to optimizing treatment efficacy and preventing side effects. In this review, we will examine the major classes of immunotherapies and biomarkers that can be utilized to determine response to treatment, treatment resistance, side effects, and different factors influencing treatment effectiveness. Also, special emphasis is placed on recent innovations in delivering mRNA through nanoparticle technology, novel advances, and challenges in cancer immunotherapy.

## 2 Approaches to cancer immunotherapy

Physiologically, the immune system has arranged specific immunological barriers (immune checkpoints) to prevent immune cells from attacking or destroying robust cells, but to treat cancer, these barriers need to be trespassed, and that is when ICIs (immune checkpoint inhibitors) come into play as part of cancer immunotherapy (Patwekar et al., 2024; Mamdani et al., 2022). The T-cell surface includes inhibitory immunoreceptors such as Cytotoxic T-lymphocyte associated protein-4 (CTLA-4), Programmed Death-1(PD-1), Lymphocyte Activation Gene 3 (LAG3), T cell immunoglobulin and mucin-domain containing-3 (TIM3), T-cell immunoreceptor with immunoglobulin and ITIM domains (TIGIT), and B and T lymphocyte attenuator (BTLA) (Lu and Tan, 2024).

### 2.1 Immune checkpoint inhibitors

**FIGURE 1:**
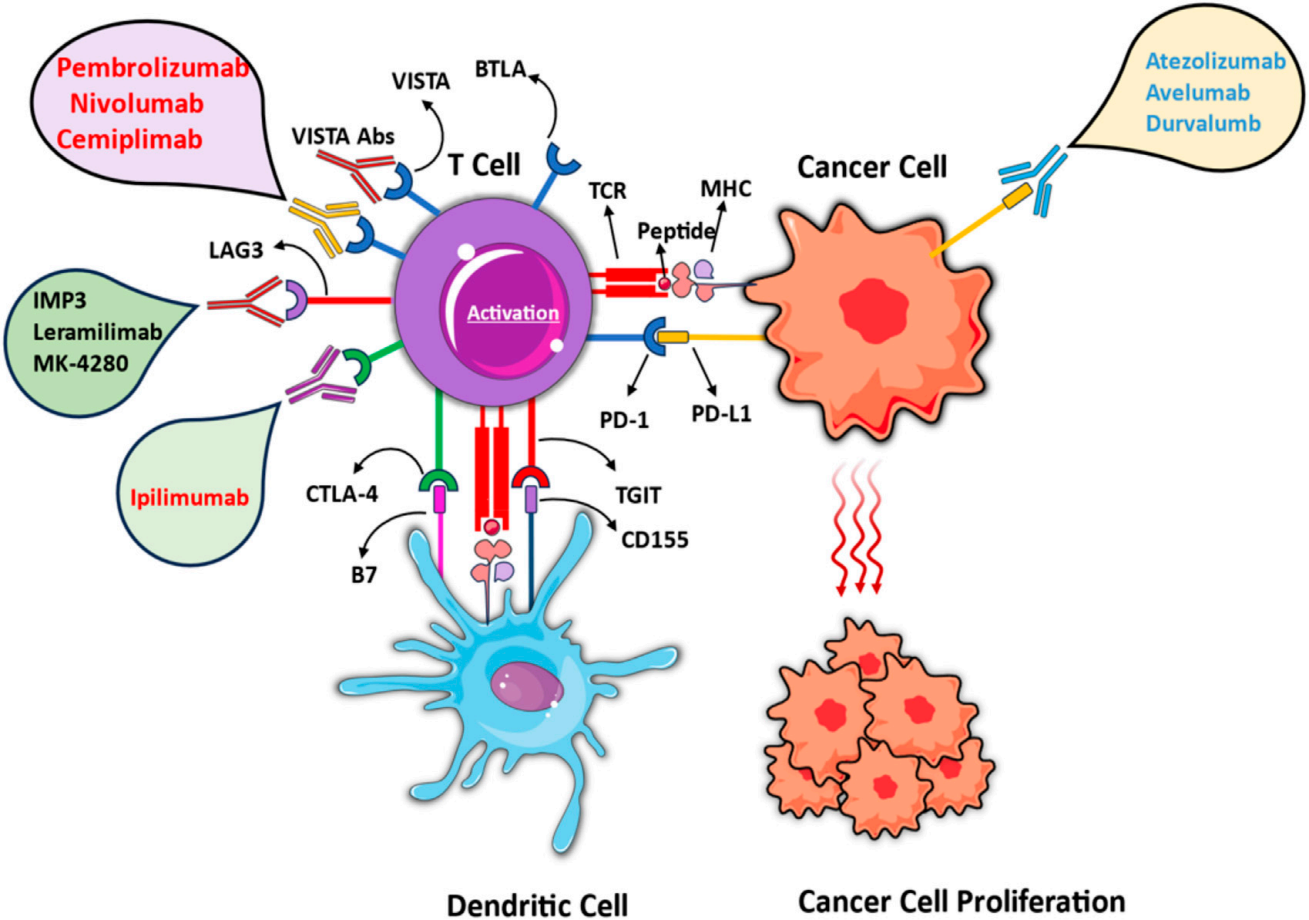
Mechanisms of immune checkpoint inhibition in cancer immunotherapy. Different mechanisms of immune-checkpoint inhibition in cancer immunotherapy, emphasizing the regulation of T cell activation through interactions with cancer cells and dendritic cells. Pembrolizumab, Nivolumab, Cemiplimab (PD-1), and Atezolizumab, Avelumab, Durvalumab (PD-L1) block PD-1/PD-L1 Axis. Ipilimumab obstructs CTLA-4/B7 interaction. IMP3, Lerapamilimab, MK-4280 target emerging checkpoint pathways like LAG3. The figure contains modified images from Servier Medical Art, licensed under Creative Commons attribution 4.0 Unported License.

ICIs are a promising class of cancer immunotherapy with numerous pre-FDA-approved treatment strategies. Their mechanism of action is based on inhibiting negative regulatory markers from T-cells, which are checkpoints responsible for their regulation (Figure 1). Activated T cells induce the inhibitory receptor CTLA4, followed by activation of PD-1, which binds to the stimulatory ligands B7-1, B7-2, and PD-L1 (programmed death ligand 1) or PD-L2 (programmed death ligand 2). The ligands are next presented to CD4^+^, CD25^+^ regulatory T cells (Tregs), myeloid cells, and antigen-presenting cells (APCs), to suppress cytotoxic T-cell activation, which allows tumor cells to grow and mitigate immune system activation (Buchbinder and Desai, 2016). It was hypothesized that by targeting ICIs, cancer cell growth can be controlled via the activation of primed cytotoxic T cells (Wang et al., 2022). As a result of adaptive resistance, traditional targets have demonstrated limited efficacy thereby paving the path for the discovery and investigation of novel checkpoint molecules including NKG2A (Natural killer cell receptor 2A) ligands, TIM3, Galectin 3, B7-H6 ligands, and so on that can improve treatment outcomes.

ICIs against CTLA-4 and PD-1, and combination therapy for anti-CTLA4 and anti-PD-1 have been approved by FDA, while there are ongoing trials of other candidates such as TIGIT, Tim3, LAG-3, Ig T-cell activation suppressor (VISTA), and IDO1 (Indoleamine 2,3-Dioxygenase 1)/IDO2 (Indoleamine 2, 3-Dioxygenase 2)/TDO (Tryptophan 2,3-Dioxygenas), CD27/CD70, CD39/73, HVEM (Herpesvirus entry mediator)/BTLA and B7-H3 (B7 Homolog 3 Protein, CD276) (Ziogas et al., 2023; Peterson et al., 2022). Clinical trials data of immune checkpoint inhibitors targeting corresponding receptors has been reported and summarized in Table 1.

**TABLE 1:** List of Immune Checkpoint Receptors and their corresponding Immunotherapeutics currently in Clinical Trials.

Immune checkpoint receptor	Function	Inhibitor/Antibody	NCT number
PD-1	Inhibits T cell activity and maintains peripheral tolerance, preventing autoimmunity	Pembrolizumab	NCT04524269
		Nivolumab	NCT02657889
		Cemiplimab	NCT03044730
		Toripalimab	NCT04000403
		Sintilimab	NCT03629925
PD-L1	PD-L1 ligand binds to PD-1 receptor and activates T-cell cytotoxicity against cancer cells	Atezolizumab	NCT03526887
		Durvalumab	NCT04294810
		Avelumab	NCT03971409
		Tislelizumab	NCT03384433
		Envafolimab	NCT02960282
CTLA-4	Controls T cell activity and mediates crucial maintenance of peripheral tolerance	Ipilimumab	NCT03520959
		Tremelimumab	NCT03470922
		Quavonlimab	NCT03794440
		AGEN1884	NCT02694822
LAG3	Negative regulator of T-cell activation	Relatlimab (BMS-986016)	NCT04552223 NCT01968109 NCT02935634 NCT02996110 NCT02750514
		REGN3767	NCT03005782
		IMP321	NCT02614833
		LAG525	NCT03499899
		MK-4280	NCT03598608 NCT02720068
TIGIT	Negatively modulates NK cells and T-cells activity; restricts T-cells function by elevating the secretions of IL-10 from dendritic cells through reverse CD155 signaling	MTIG7192	NCT03563716
		BMS-986207	NCT05005273
		OMP-313M32	NCT05715216
		MK-7684	NCT05224141
TIM-3	Suppresses Cytotoxic T Lymphocytes and effector Th1 cell function	TSR -022	NCT02817633
		MGB453	NCT02708268
		BGBA425	NCT03744468
		R07121661	NCT03708328
BTLA	Interaction with HPEM leads to suppression of immune responses and dampen T cell activity	SHR-1701	NCT03990233
		JTX-8064	NCT04669899

#### 2.1.1 Cytotoxic T-lymphocyte associated protein-4 (CTLA4)

CTLA4 is an immune regulator from the Ig superfamily. CTLA4 is expressed lowly on the basal level by Treg cells and is inducible post-activation by an antigen. It shares a similar structure, and biochemistry with CD28, and resides in the common region of chromosome 2, 2q33.2 (Babamohamadi et al., 2024). CD28 and CTLA4 create membrane-bound homodimers from an extracellular Ig-like domain, a transmembrane region, and a cytoplasmic tail. Post activation, CTLA4 is controlled by lipopolysaccharide-responsive and beige-like anchor protein (Waldman et al., 2020). However, CD28 and CTLA4 are functionally antagonistic, i.e., CTLA4 inhibits T cell activation and proliferation, whereas CD28 provides co-stimulatory signals for their activation (Gardner et al., 2014).

CTLA4 has several mechanisms for inhibiting T cell activation, including targeting CD28 through intracellular vesicles that release CTLA4; by reorganizing the cytoskeleton to prevent the formation of T cell-APCs immune conjugate; through internalization of CD28 specific ligands thus disrupting their binding; and by using phosphatases such as protein phosphatase 2A (an inhibitor of extracellular signal-regulated kinase) and SH2 domain-containing tyrosine phosphatase 2 (inhibits phosphorylation of the CD3 ζ-subunit of T cell receptor (TCR) (Rudd et al., 2009). As a result, T cells avoid activation through inactivation of activator protein 1 (AP-1), nuclear factor-κB (NF-κB), and nuclear factor of activated T cells (NFAT) (Atsaves et al., 2019).

ICIs targeting CTLA4 effectively enhance immunity, activate T cells, induce immunological memory, as well as upregulate the T cell response to tumor-associated neoantigens to kill cancer cells and attack Treg cells in tumors (Seidel et al., 2018). Anti-CTLA4, Tremelimumab, inhibits the action of CTLA4 and blocks the T-cell suppression, and also stimulates the development of cytokines like IL-2 and interferon-gamma (IFN-γ) thereby improving the responsiveness of the immune system to fight against different cancers like renal cancer, melanoma and non-small cell lung cancer (NSCLC) (Fu et al., 2024). Another anti-CTLA4 antibody is Ipilimumab, the first ICI approved by the FDA in 2011 for advanced melanoma (Lipson and Drake, 2011). Alteration in the IFN-γ pathway, along with defects in genes like JAK2, IRF1, IFNGR1, and IFNGR2, and also the amplification of IFN-γ inhibitory genes like PIAS4 and SOCS1, contributes to enhancing CTLA-4 functions thereby causing obstruction and resistance of anti-CTLA4 therapy (Mojic et al., 2017).

#### 2.1.2 Programmed Death-1 (PD-1)

A type 1 transmembrane glycoprotein from the immunoglobulin superfamily, PD-1 shares a similar amino acid sequence with CTLA4 and CD28. PD-1 inhibits T cell activation in peripheral tissues, targets inhibitory intracellular-signaling cascade in effector T cells and Treg cells, and facilitates T cell exhaustion to create a tumor microenvironment (TME) and promote tumor division. Moreover, PD-1 inhibits protein kinase B through protein phosphatase 2A and decreases phosphoinositide 3-kinase (PI3K) activity (Riley, 2009). PD-1 inhibition enhances antitumor immunity, activates T-cell cytotoxicity against tumor cells, releases inflammatory cytokines and cytotoxic granules, and limits metastasis (Darvin et al., 2018). Pembrolizumab and nivolumab are human-derived IgG4 second-generation ICIs, that are FDA-approved and have displayed outstanding results in clinical trials for the treatment of refractory and unresectable melanoma (Ai et al., 2020). The treatment results demonstrated that pembrolizumab led to an improvement in progression-free survival. The first approved PD-L1-targeted humanized IgG4 antibody atezolizumab has been approved for the treatment of urothelial carcinoma, and FDA has also recently approved avelumab and durvalumab (Massari et al., 2018). Reduced infiltration of CD8 T-cells into tumors can cause resistance to PD-1 therapy due to the blockade of the PI3K/AKT pathway. Besides this, limitations to anti-PD-1 therapy include mutations in the JAK1/2 pathway, loss of IFN-γ signaling, and microbial imbalance in the body (Vaddepally et al., 2020). Although the combination therapy with anti-CTLA4 and anti-PD-1 has shown promise, increased toxicity remains a challenge that limits current clinical use (Das et al., 2015).

#### 2.1.3 T-cell immunoreceptor with immunoglobulin and ITIM domains (TIGIT), Ig T-Cell activation suppressor (VISTA), and T-cell immunoglobulin and mucin-domain containing-3 (TIM-3)

TIGIT is another target for the ICIs, derived from the poliovirus receptor linked nectin family of proteins, which inhibits CD8^+^ and CD4^+^ T cells, Tregs and natural killer (NK) cells, and thereby activates immunity through the CD226-PVR pathway (Gorvel and Olive, 2020). TIGIT structurally comprises an Ig extracellular variable domain, a type-I transmembrane domain, and a cytoplasmic tail with inhibitory motifs, ITIM, and tyrosine-like Ig tail motifs (Yu et al., 2009).

TIGIT binds to ligands CD155 and CD112, which are regulated by tumor cells and APCs in the tumor environment (Chauvin and Zarour, 2020). Elevated TIGIT activity through interaction with CD155 affects tumor-specific CD8^+^ T cells, limiting TCR-induced p-ERK signaling, suppressing IFN-γ production, and NK-mediated cytotoxicity. Blocking TIGIT binding to CD155 eliminates NK cell dysfunction and activates anti-cancer activity (Chauvin et al., 2020). Many monoclonal antibodies targeting TIGIT have already been developed, including tiragolumab, vibostolimab, and etigilimab, which can be utilized as single therapy or in combination with anti-PD-1 or anti-PD-L1 agents. This combination strategy has been proven to have a superior effect (Rotte et al., 2021).

VISTA is used as a targeted therapy because VISTA interacts and binds to P-selectin glycoprotein ligand-1 (PSGL-1) and keeps naive T cells and myeloid cells in an inactive state, reprograming macrophages into an immunosuppressive phenotype in TME, especially under low-pH and hypoxic conditions. Antibodies against VISTA including SNS-101 are also pH-selective and can inhibit cancer growth (Yuan et al., 2021; Thisted et al., 2024).

TIM-3 is a co-inhibitory molecule expressed on CD4^+^ T cells, CD8^+^ T cells, Tregs, and leukemic cells. Tim-3 is a transmembrane inhibitory protein consisting of an amino-terminal variable Ig domain called the V domain and five non-canonical cysteines, a mucin stem, a transmembrane domain, and an inhibitory cytoplasmic tail. Ligands binding to the V domain include HMGB1 (high-mobility-group box 1), CEACAM1 (carcinoembryonic antigen-related cell adhesion molecule 1), phosphatidylserine, and Galectin-9 (Wolf et al., 2020). TIM-3 cell expression during cancer affects the formation of immune cell suppressive TME, induction of NK cells, inhibits cytokine secretion, IFN-γ, and Th1-mediated responses. While binding to HMGB1 leads to impaired activation of DCs through galectin-9, TIM-3 induces apoptosis in TIM-3-positive cytotoxic T cells. Therefore, inhibition of TIM-3 interaction with ligands prevents T-cell depletion and exhibits antitumor activity (Das et al., 2017).

#### 2.1.4 LAG-3 (lymphocyte activation gene-3 CD223)

LAG-3 is a co-inhibitory receptor and member of the type I transmembrane protein of the Ig superfamily expressed on the surface of CD4^+^ and CD8^+^ T cells, NK cells, and Treg cells. Major ligands for LAG-3 include MHC-II (Major Histocompatibility complex-II), fibrinogen-like protein 1 (FGL-1), Galectin-3 (Gal-3), and LSECTin (Borgeaud et al., 2023).

LAG-3 structurally comprises an extracellular domain with four Ig-like domains similar to the CD4^+^ receptor. LAG-3 inhibits T-cell proliferation and mediates activation of effector T cells by interfering with the CD4-MHC-II interaction and through interaction with Gal-3 or FGL-1. Therefore, LAG-3 blockade achieves antitumor activity dependent on CD4^+^ and CD8^+^ T cells (Wang et al., 2019). Relatlimab is an IgG4 monoclonal antibody that has shown successful blockade of LAG-3 and its interaction with ligands, demonstrating improved survival, and antitumor activity (Thudium et al., 2022).

#### 2.1.5 IDO1 (Indoleamine 2,3-Dioxygenase 1)/IDO2 (Indoleamine 2,3-Dioxygenase 2)/TDO (tryptophan 2,3-Dioxygenas)

IDO1, IDO2, and TDO are a class of immunoregulatory enzymes involved in suppressing the T-cell immune response through catabolism of tryptophan (which can cause cell cycle arrest in deficiency and susceptibility to Fas-mediated apoptosis) to kynurenines (which abrogate CD8^+^ T-cell cytotoxicity) (Merlo et al., 2020). These enzymes are normally expressed in various cells, including TDO expression in the liver and IDO expression in epithelial, immune, and endothelial cells (Biswas and Stuehr, 2023). In cancerous cells, IDO suppresses cytokine and granular cytotoxic protein production, thereby mediating loss of functionality in lysis tumor cells besides the decreased number of cytotoxic T cells in TME. However, for regulation via the tryptophan-kynurenine pathway, blockade of IDO1/IDO2/TDO is required (Meireson et al., 2020).

Another interesting target to add to the medicinal arsenal against cancer is an enzyme called ART-1 which is expressed on tumor cells. It has the power to tweak the receptor present on immune cells in a way that can induce cell death and thereby thwart T-cells from fighting against the tumor. Preclinical studies conducted in mice induced with NSCLC have revealed astounding results where it was evident that ART-1 modifies the P2RX7R receptor present on CD8 T cells triggering a signaling pathway that eventually leads to CD8 T cells’ death. To combat this, scientists have developed an antibody that inhibits ART-1 and restores anti-tumor immune activity to slow down or stop tumor proliferation. NR4A1 is a recent addition to the target toolkit for immunotherapy and its role is to suppress the anti-tumor immune system for a prolonged period. Wang et al, have utilized proteolysis-targeting chimera (PROTAC, NR-V04) to degrade NR4A1. *In vitro* studies reveal that it takes hours to degrade NR4A1, and this degradation effect is long-lasting. Mechanistically, NR-V04 downregulates monocytic myeloid-derived suppressor cells and instigates tumor-infiltrating B cells and effector memory CD8^+^ T cells.

### 2.2 CAR T-Cell therapy

Adoptive cellular therapies encompass *ex vivo* activation of a patient’s immune cells, which are then transferred back to fight cancer. These include 3 major therapies, i.e., CAR-T cells, tumor-infiltrating lymphocytes (TILs), and genetically modified TCR therapies. Among them, Chimeric Antigen Receptor T-cell therapy (CAR-T) cell therapy has revolutionized cancer treatment, producing promising results in certain subtypes of B-cell leukemia, with over 5 products approved by the FDA (Fang et al., 2022). The treatment entails separation of a patient’s peripheral blood mononuclear cells (PBMCs) by leukapheresis or density-gradient centrifugation, following which CD8^+^ and CD4^+^ regulatory T cells are activated through exposure to nano-matrix-, plate- or beads bound anti CD3 and anti-CD28 antibodies (Vormittag et al., 2018). These isolated T cells are genetically engineered by DNA-encoded permanent impression into the T cell genome. The modified CAR-T cells replicate the typical T cell signaling pathways when they come into contact with cancer cells, proliferating and initiating an anti-tumor response. A recognition domain built into the CAR’s architecture aims to target tumor-associated antigens (TAAs) present on cancer cells. The CAR-T cell and the cancer cell form a non-traditional immunological synapse upon antigen identification, which triggers complex biological processes such as clonal expansion and receptor clustering. As a result, target cells are destroyed by processes like apoptosis and exocytosis (Ruf et al., 2024). Additionally, activated CAR-T cells release cytokines including IL-2, IL-6, and IFN-γ, which attract and activate immune cells like macrophages, natural killer (NK) cells, and more T cells, thus promoting tumor suppression (De Marco et al., 2023).

First-generation CARs activated T cells independently of the major histocompatibility complex, presenting specific target antigen via the extracellular single-chain variable fragment antibody domain. The hinge region further connects the extracellular and intracellular domain through the transmembrane domain. Whilst the first-generation CARs comprise a CD3ζ signaling domain that mimics the T-cell receptor signaling pathway, facilitating low cytotoxicity and anti-tumor effectiveness, the second-generation CARs include both CD3ζ and co-stimulatory domain CD28, that enhances T cell proliferation (Tomasik et al., 2022). The third-generation CARs comprise of two co-stimulatory domains, whereas the fourth and fifth generation are enriched by additional signaling domains including 4-1BB or interleukin 12 cytokines. A key upgrade in fourth and beyond generation is the genetic manipulation via T cell redirected for antigen-unrestricted cytokine-initiated killing (TRUCKs), which mediates extensive cytokine release and modulation of immunological and vascular tumor environment, enhancing anti-tumor activity and minimizing off target toxicity (Chen T. et al., 2024).

CARs consist of antigen-binding domain associated with T cell receptor signaling domains and co-stimulatory molecules to acquire specificity through polymorphic α- and β-glycoprotein chains with antigen-binding and conserved domains to bind to CD3, CDγ, CDδ, CDε, and CDζ. Alternatively, lymphocytes derived from the patient’s peripheral blood mononuclear cells (PBMC) express the desired TCR or CAR (Chang and Chen, 2017). For example, ZUMA-1 (axicabtagene ciloleucel or axi-cel, which is the CD28 costimulatory domain) and JULIET (tisagenlecleucel or tisa-cel, which is the 4-1BB co-stimulatory domain) act as anti-CD19 CART (CAR19) in relapsed or refractory diffuse large B-cell lymphoma and multiple myeloma, as well as non-Hodgkin’s B-cell lymphoma (Frigault and Maus, 2020). Axicabtagene ciloleucel, brexucabtagene, autoleucel, lisocabtagene, maraleucel, and tisagenlecleucel have been approved by the FDA, while idecabtegene, vicleucel and ciltacabtagene, autoleucel are approved as B-cell maturation antigen (anti-BCMA) CAR-T (Chekol Abebe et al., 2022). Moreover, other immune cell therapies such as CAR-NK cells, or CAR-macrophages therapies are being developed but have not yet been approved by the FDA (Meng et al., 2021). Hematological malignancies are the most effective targets for CAR-T cell therapies compared to solid tumors due to the absence of a thick extracellular matrix (ECM).

Despite ground-breaking results in the clinical management of malignant tumors, CAR-T cell therapy faces obstacles concerning wider use and efficacy. The short half-life and less effectiveness of CAR-T cells are major concerns associated with CAR-T cell therapies, leading to eventual signalling worn out despite an initial strong stimulation. Low CAR-T cell persistence or the unregulated proliferation of certain subclones (antigen-negative cells) might lead to the development of resistant tumors or relapse (Bouziana and Bouzianas, 2021). Newer generations of CARs have been developed to counter this, as mentioned above. Vast evidence in clinical data, increased stability, and more mature technological processes have placed second-generation CARs at the forefront of cancer treatment. Solid tumors offer a variety of challenges, such as minimal T cell infiltration, unfavorable tumor microenvironment, and low levels of antigen expression in healthy tissues, which may lead to off-target effects for which some counter-strategies have been identified in recent times (Flugel et al., 2023). Significant hurdles to CAR-T cell entry in solid tumors include the extracellular matrix, a hostile milieu, and the variable expression of TAAs. Hostile environments like hypoxia, acidity, and immunosuppressive cells make it challenging for CAR-T cells to perform their job, especially in patients with solid tumors (Bot et al., 2015). Novel CAR-T armed with a synNotch receptor that upregulated IL-2 expression when in contact with specific antigens, reported greater tumor-infiltration and better efficacy (Allen et al., 2022). Development of diverse fourth-generation CARs is paving the way to improve control, specificity, and effectiveness in targeting cancer cells (Tang et al., 2023a). Different types of CAR-T cells and their mechanisms are as follows:

A. Logic-gated CAR-T Cells: Operated as Boolean cells. As the name suggests, operator techniques AND, IF-THEN, and OR increase efficacy by expanding the target cell pool while decreasing off-tumor damage. Cells that exhibit AND/OR mechanism are called Dual CAR-T cells and those that exhibit IF/THEN are Conditional expression CAR-T cells.
B. Controlled CAR-T Cells: Due to their ability to alter the activity, clinicians can regulate how these affect non-malignant cells by mechanisms like switchable CARs, inhibitory CARs, and suicide CAR-T cells. Switchable CARs (also known as Universal or Split CARs) allow for the substitution or alteration of their ligand-binding domain (LBD). This lowers antigen escape and increases target specificity by enabling CAR-T cell rerouting towards distinct cancer cell subpopulations. Inhibitory CARs (iCARs) reduce off-tumor toxicity and avoid harm to non-cancerous tissues by co-expressing an inhibitory antigen that prevents CAR-T cell activation in the presence of healthy cells. Suicide CAR-T Cells incorporate an “off-switch” that enables practitioners to cause CAR-T cells to undergo apoptosis, halting their function when necessary.
C. ECM-Degrading CAR-T Cells: These CAR-T cells can break down the extracellular matrix (ECM) in solid tumors due to their acquired ability to express enzymes like heparanase (HPSE) through genetic engineering. This makes them better at combating tumors rich in extracellular matrix (ECM) by penetrating the thick tumor stroma.
D. Universal CAR-T Cells (UCARTs): These CAR-T cells are allogeneic. Their purpose is to reduce immunogenicity and avoid GVHD (graft-versus-host disease).

### 2.3 Cancer vaccines

Cancer vaccines constitute another modality of the immunotherapeutic approach, based on the principle of enhancing and building the patient’s T-cell immunity, which strengthens the immune response and destroys TAAs. Cancer vaccines are fabricated to target TAAs (neoantigens, or neoepitopes) that are generally expressed on cancer cells. The therapeutic pathway followed by most cancer vaccines is the stimulation of cell-mediated responses, like those from CTLs that can minimize tumor burden (Vigneron, 2015; Türeci et al., 2016). In this way, a durable immunity is created and directed strictly to target cancer cells (Le et al., 2022). Currently, there are 4 different categories of cancer vaccines, namely, cell-based, peptide-based, nucleic-acid-based, and viral-based vaccine (Elsheikh et al., 2023). In 2010, the FDA approved the first cancer vaccine sipuleucel-T (Provenge) for prostate cancer treatment. Sipuleucel-T is a dendritic cell (DC)-based vaccine that builds immunity against prostatic acid phosphatase and activates antigen-specific T cells (Cheever and Higano, 2011). The allogeneic tumor cell vaccine uses the patient’s cancer cells to produce the vaccine and injects irradiated tumor cells together with an adjuvant, as well as genetically alters the cancer cells in such a way as to incorporate additional functions, including the production of cytokines, costimulators or granulocyte-macrophage colony-stimulating factor (GM-CSF) (Hammerstrom et al., 2011).

DC vaccines are successful because cancerous DNA or RNA, viruses, proteins, or peptides can be loaded on DCs, while the DC surface expresses receptors for antigen binding (Igarashi and Sasada, 2020). These antigens are presented to immune cells, which activate the immune system. In contrast, protein-peptide vaccines stimulate the immune response against specific antigenic epitopes derived from proteins or peptides of cancer cells. APCs will recognize those peptides or proteins and capture them, and then T cells will induce an immune response (Purcell et al., 2007). Thus, antigenic epitopes derived from TAAs are capable of binding to HLA, while antigens derived from cancer-specific gene mutations are used, which will be more specific in detection (Zhao et al., 2021). Vaccines generally comprise short-chain peptides (SPs) restricted to MHC class I, which reduces the success in activation of MHC class II-restricted cells, such as helper T cells. Therefore, new generation vaccines consisting of long-chain peptides with epitopes for both CTL and helper T cells and capable of activating MHC I and MHC II are under development for more successful immune responses and clinical applications (Buonaguro and Tagliamonte, 2020).

Nucleic acid-based cancer vaccines have also shown activation of MHC class I-mediated CD8^+^ T-cell response through activation of humoral and cellular immunity. They activate the immune system through multiple epitopes (Shi et al., 2022). RNA-based vaccines have several advantages over DNA-based vaccines as they don't incorporate into the genome, prevent carcinogenicity, and are functional in the cytoplasm, while DNA vaccines need to be transferred to the nucleus. To transfer nucleic acids, viral vectors are used (Leitner et al., 1999).

Oncolytic virus immunotherapy attacks tumor cells directly and stimulates immune responses. Vectors for specific genes and tumor antigen expression, including adenovirus, poxvirus, alphavirus, and herpes simplex virus, are utilized abundantly, either naturally occurring or genetically engineered (Kaufman et al., 2015). Adenoviruses are preferred because of their ability to transduce dividing and nondividing cells, while herpes simplex virus type 1 is prominent due to its oncolytic properties and GM-CSF expression (Matsunaga and Gotoh, 2023). The virus is made benign and aims to kill cancer cells without affecting normal, healthy cells. The destruction of cancer cells occurs through the introduction of a modified virus that targets cancer in two ways: Direct and Indirect (Javid et al., 2023).

The direct mechanism causes oncolysis through cell infection with an oncolytic virus and death. In the indirect mechanism, the virus induces an anti-inflammatory response and the release of microorganism-associated molecular patterns, danger-associated molecular patterns (DAMPs), thus producing APCs, DCs, and macrophages in TME (Hofman et al., 2021). Moreover, captured tumor antigens are released through oncolysis and migrate to regional lymphoid structures to activate tumor-specific T cells to destroy cancer cells by production of perforin and granzyme B, and their recognition by T cells through expression of MHC molecules and presentation of tumor antigens on the surface of cancer cells (Del Prete et al., 2023). T-VEC (Imlygic), is an FDA-approved viral-based vaccine that uses herpes simplex virus as a vector and has displayed strong anti-cancer immunity (Conry et al., 2018). However, TAAs are expressed not only in tumor cells, but also in normal cells with gene mutations caused by carcinogenesis. Therefore, a vaccine targeting TAAs may induce autoreactive immune responses, high toxicity, and autoimmunity (Kaczmarek et al., 2023).

Microbes can potentially affect the safety and efficacy of cancer immunotherapy by regulating the host’s immune responses. They impact tumorigenesis by secreting certain toxins and creating an immunosuppressive ecosystem. Lipopolysaccharides (LPS) and short-chain fatty acids (SCFA) are microbe-derived components that expend anti-tumor activity. One such SCFA is butyrate, which inhibits histone deacetylases and subdues tumor progression. Emerging research proposes four mechanisms by which gut microbiota modulates responses to immunotherapy: 1) Enhanced anti-tumor immune response due to stimulated T cell activity in response to microbial antigens. 2) cross-interaction with TSA (Tumor-specific antigens). 3) Induction of immuno-stimulatory or anti-inflammatory response by attaching to Pattern recognition receptors (PRRs). 4) Through metabolites that regulate systemic reactions on the host. Diet is known to direct the intestinal microbiota towards modulating the host immune response to cancer immunotherapy. Fibre-rich diet flourishes the microbial ecosystem, thereby promoting SCFAs synthesis.

### 2.4 mRNA vaccines

mRNA vaccines are an upcoming class of therapeutics broadly treating cancer, cardiovascular, cerebrovascular, infectious, metabolic genetic diseases, and autoimmune disorders. Almost two decades ago, the potential of mRNA to combat cancer was explored, and it was not just restricted to ideation but also backed with published proof-of-concept studies (Conry et al., 1995; Boczkowski et al., 1996). Boczkowski et al were the first ever research group to showcase the feasibility of electroporation of dendritic cells with ovalbumin encoding mRNA to initiate an immune response against tumor antigens in melanoma mice models (Boczkowski et al., 1996). A similar study proved the efficacy of a formulation containing Trimix, which is a fusion of mRNA-encoded adjuvants (CD70, CD40L, and constitutively active TLR4) electroporated with mRNA in pre-clinical studies (Bonehill et al., 2008). The reason for this enhanced efficacy was suspected to be improved activation of dendritic cells, and switching of CD4^+^ T cell phenotype from T regulatory cells to T helper 1-like cells (Bonehill et al., 2008; Wilgenhof et al., 2011). A related report published data showing tumor regression in 27% of stage III or IV melanoma patients immunized with the above-mentioned formulation (Wilgenhof et al., 2013). Surprisingly, a tailored mRNA vaccine for metastatic melanoma developed by BioNTech showed no identifiable lesions on radioactive imaging and was found to be recurrence-free even after 23 months of intra-nodal delivery. However, lack of sufficient data necessitates further research to consider mRNA vaccines for melanoma as trustworthy (Sahin et al., 2017).

These exciting results have paved the way for multiple clinical trials that exploited DC (dendritic cells) vaccines for treating various cancers including metastatic prostate cancer, metastatic lung cancer, renal cell carcinoma, brain cancers, melanoma, acute myeloid leukaemia, and pancreatic cancer (Mitchell et al., 2015; Batich et al., 2017). mRNA pulsed DC vaccines developed using mRNA copies of GBM (Glioblastoma) in patients increased progression-free survival up to 2.9 times greater than the control (Vik-Mo et al., 2013). The survival time was further prolonged due to increased bilateral DC migration on pre-conditioning the immunization site with potent recall antigen, namely, tetanus/diphtheria toxoid (Mitchell et al., 2015). Hanna J Khoury et al performed a study that resulted in encouraging outcomes to treat acute myeloid leukemia, where an intradermally injected mRNA encoding human telomerase reverse transcriptase ensured complete remission in 11 out of 19 treated patients (Khoury et al., 2017; de Lima et al., 2014). For renal cell carcinoma, anti RCC-DC based mRNA vaccine was intradermally administered to generate specific immune response that resulted in prolonged survival of RCC patients (Rittig et al., 2016).

Recently, lipid and polymeric nanoparticle technology have emerged as robust delivery systems owing to their versatile nature. Lipid nanoparticles have garnered attention as convenient mRNA delivery agents. This wide usage is attributed to its invaluable characteristics like improved stability, safety, and transfection efficiency (Qin et al., 2022). They are comprised of 4 elements, namely, cholesterol acting as a stabilizer, phospholipids that support the lipid bilayer geometry, a lipid-linked PEG that increases half-life of the delivery systems, an ionizable cationic lipid (e.g., 1,2-di-O-octadecenyl-3-trimethylammonium propane [DOTMA]) that causes self-assembly into particles of size as small as 100 nm, and initiates endosomal mRNA release into the cytosol (Qin et al., 2022). Some commonly used lipids include N, N-Dimethyl-2,3- bis [(9Z, 12Z)-octadeca-9,12-dienyloxy]propan-1-amine (DLinDMA) and N1, N3, N5 - tris(3-(didodecylamino) propyl)benzene-1,3,5-tricarboxamide (TT3).

The positive charge carried by the lipid at a particular pH is responsible for the encapsulation of negatively charged RNA through electrostatic interactions, and forms something called lipoplex (Zeng et al., 2022; Liang et al., 2021). Dr. Pieter Cullis, a pioneer in this field, discovered that encapsulation of antigen in liposome nanoparticles showed greater immunogenicity (Liang et al., 2021; de Jong et al., 2007).

Yang et al., synthesized lipid nanoparticles containing cholesterol and a modified cationic peptide DP7. This resulted in enhanced *in vivo* cellular uptake of mRNA, and also induced activation of dendritic cells (Qin et al., 2022). Phua et al., fabricated a mesoporous-silica nanoparticle to condense both mRNA and RNA-activated protein kinase inhibitor C16. This vaccine was found to inhibit tumor growth significantly. As of June 2021, it was reported that every SARS-CoV-2 mRNA vaccine that received approval for clinical use was delivered using lipid nanoparticles (Chaudhary et al., 2021).

Another functional material-based mRNA delivery approach leverages the potential of polymeric materials such as polyamines (e.g., polyethyleneimine) and dendrimers (e.g., polyamidoamine), which protect mRNA from degradation and promote intracellular uptake. One plausible mechanism by which polyplexes (complex of cationic polymers and mRNA) evade endosomal uptake is proposed to be the proton buffering by the cationic polymer ensuing osmotic swelling, followed by endosomal rupture, also known as the proton sponge effect (Chaudhary et al., 2021).

Back in 1987, polylysine (PLL) came into limelight as the first ever cationic polymeric vector to effectively transfect plasmid DNA. Later, polymeric carriers like spermine, polyurethane, etc., were scouted for their ability to safely deliver mRNA. The use of PEI is restricted due to its toxicity profile and non-biodegradable nature, thereby making room for further developments. Jere et al., have performed some studies, to deduce that introducing polysaccharides, and encapsulation of mRNA with PEI into neutrally charged liposomes has diminished non-specific adhesion (Jere et al., 2009). Xiyu et al., designed PEI-g-PEG using multiple PEG terminal groups with varying PEG grafting degrees to deliver mRNA with adequate potency (Ke et al., 2020). This mode of delivery has been further advanced by incorporating some additional components, such as biodegradable units and lipid chains to aid in improving the stability of the formulation. For instance, Kaczmarek et al., successfully synthesized a biocompatible polymer, poly (β-amino esters), capable of delivering mRNA that could target lung endothelial cells and immune cells (Kaczmarek et al., 2018). A slight modification to this was made by Kowalski et al, where mRNA was injected intravenously into the spleen using a novel PCL-PBAE construct (Kowalski et al., 2018).

Similarly, polyamidoamines, the hyperbranched spherical dendrimers possessing high charge density, allow satisfactory complexation with mRNA, nonetheless, it causes serum aggregation. The incorporation of disulfide linkages into the dendrimer core attenuates their toxicity issue (Chaudhary et al., 2021). Another biodegradable positively-charged polymer, charge-altering releasable transporters (CARTs), was synthesized to improve mRNA loading efficiency and certain physical properties by the phenomena of charge-neutralization intramolecular rearrangement, which aids in the release of functional mRNA and promotes protein translation in cells.

Wang et al constructed an injectable hydrogel consisting of polyethylene imine and graphene oxide to carry mRNA-encoding ovalbumin alongside adjuvant R848 that reported inhibition of tumor progression in the B16-OVA melanoma model. Cationic peptides composed of amino acids such as lysine and arginine present amino groups that electrostatically interact with phosphate structures of mRNA. A cell-penetrating peptide RALA, was explored to deliver mRNA to the immune cells. Another widely accepted delivery strategy is cationic nanoemulsions, which integrates cationic lipids like DOTAP and nanoemulsions. Hydrophobic and hydrophilic surfactants are combined to stabilize the oil phase in the aqueous environment, leading to particle generation. It is tested and proven that the addition of cationic lipids facilitates complexation with nucleic acids via electrostatic forces and thereby imparts stability to mRNA (Teixeira et al., 2017).

Researchers have designed creative mRNA delivery strategies, which include, but not limited to reactive astrocyte-derived exosomes, tetrasulfide-embedded dendritic mesoporous organosilicon nanoparticles, lipid-decorated calcium phosphate nanoparticles, nucleoside lipids, and so on (O'Brien et al., 2020; Schumann et al., 2018; Wang et al., 2020). Immunologic adjuvants play a key role in enhancing the efficacy of cancer vaccines by boosting the immune system’s response to tumor-associated antigens. The mechanism involved is activation of the innate immune system, which triggers the production of cytokines that further activate the adaptive immune response. These adjuvants, through stimulation of antigen-presenting cells (APCs) like dendritic cells, promote the tumor antigen presentation to T-cells, thereby instigating an immune response. Few examples of immunoadjuvants are Toll-Like Receptor (TLR) agonists such as Polyinosinic-polycytidylic acid that activates TLR3, CpG Oligodeoxynucleotides that stimulate TLR9, and Imiquimod (TLR7 agonist), which are responsible for eliciting Th1-biased immune responses. Other pivotal adjuvants include DDA-Saponin Complexes (saponin-based adjuvants) that promote antigen presentation and foster balanced Th1/Th2 responses. Interleukin-15 (IL-15) is a cytokine adjuvant that prolongs anti-tumor activity via activation of natural killer (NK) cells and CD8^+^ T cells (Desai et al., 2025). Overall, adjuvants are crucial in driving the progress of strong cytotoxic T lymphocytes (CTLs) responses (Ren et al., 2024).

### 2.5 Cytokine therapy

Cytokines are soluble proteins required for intercellular communication, cell differentiation and growth, and mediating innate and humoral immunity. Cytokines are activated in response to the stimulus. However, their circulation is short-term and have a limited half-life. Treatment with cytokines is based on either synergistic combination with approved drugs or their improved pharmacokinetics to increase half-life in the circulation, as well as topical administration of cytokines in the TME (Conlon et al., 2019). However, a conjugate with polyethylene glycol or a fusion protein with antibodies, Fc-domains, apolipoprotein A-I, albumin, or TGF-β (transforming growth factor-β) should be created to increase the half-life (Berraondo et al., 2019).

Cytokines upregulate TGF-β, triosephosphate isomerase, and IL-10, activation of Tregs, myeloid-derived suppressor cells, intracellular suppressors of cytokine signaling activation, and resulting in CD4^+^ activation. However, treatment with cytokines cannot directly affect the immune response to cancer cells, but only activate the immune response (Conlon et al., 2019). Therefore, “superkines” have been created using structural engineering. Moreover, applications of cytokines with cancer vaccines, ICIs, and monoclonal antibodies are being developed to enhance antibody-dependent cellular cytotoxicity, improve affinity binding, and enhance anticancer properties (Holder et al., 2022). Currently, cytokine structural engineering is focusing on the immunotherapeutic cytokines IL-2, IL-4, IL-15, and IFN (Aung et al., 2023).

Thus, the alterations of IL-2 reduced stimulation of Tregs, and reduced interaction with IL-2Rα, induced augmented expression of cytotoxic T cells, and improved antitumor responses (Xue et al., 2021). While IL-15 fused with truncated IL-15Rα activates IL-15Rα-deficient cells and forms a signaling complex to trigger proliferation and anti-apoptosis (Guo et al., 2017).

## 3 Importance of early assessment and identification of immune-related adverse events (irAEs)

**FIGURE 2:**
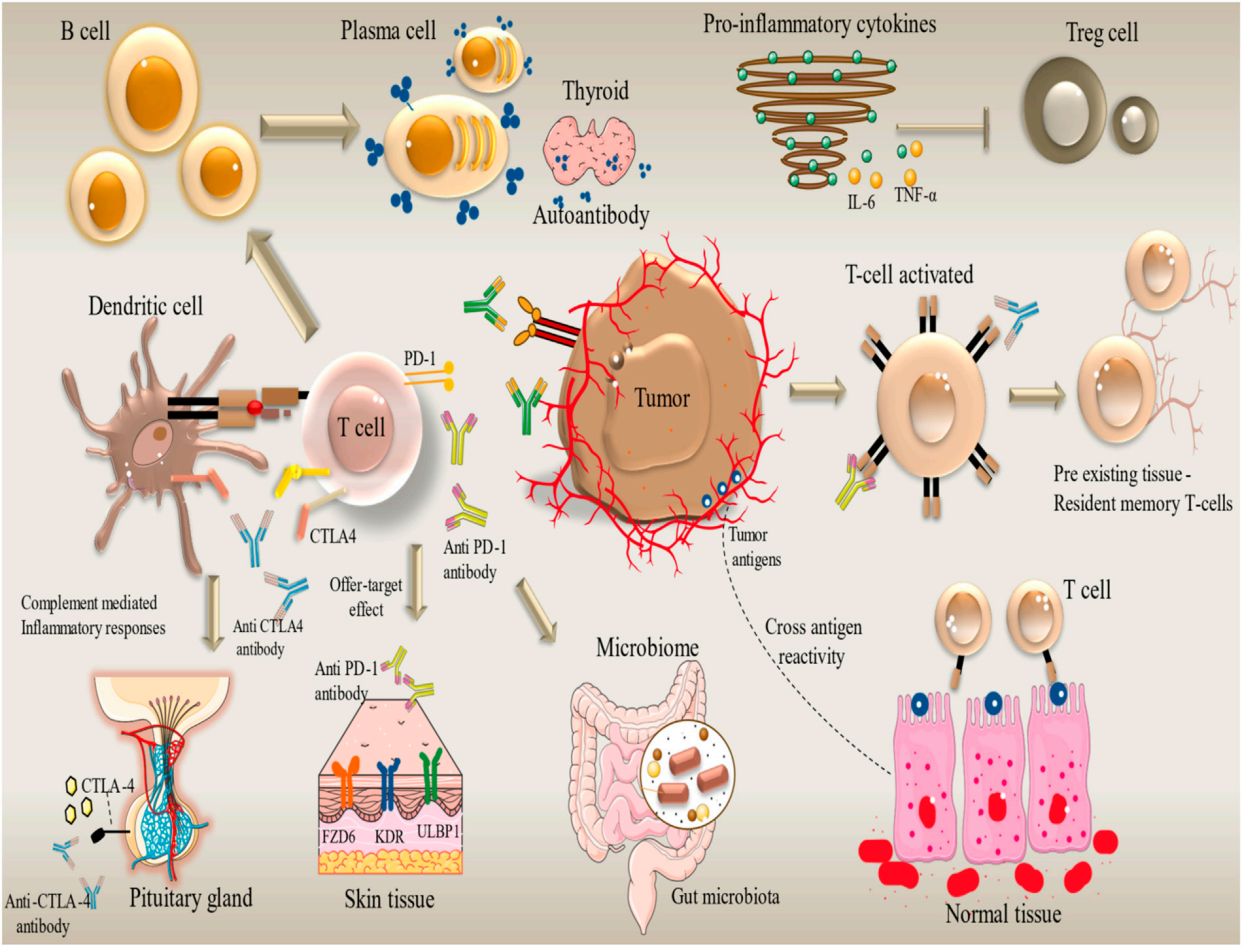
Organ-specific immune-related adverse events (irAEs) induced by immune checkpoint inhibitors in cancer therapy. irAEs Development. Immune-related Adverse Events (irAEs) can affect any organ system. Different immunotherapy drugs cause different irAEs in different organs. For example, endocrine toxicity (Nivolumab), dermatologic toxicity (Camrelizumab), hypothyroidism (Atezolizumab), and pneumonitis and hepatitis (Pembrolizumab). The occurrence of grade 3/4 irAEs is an indicator of discontinuing immunotherapy. The figure contains modified images from Servier Medical Art, licensed under Creative Commons attribution 4.0 Unported License.

Tailoring ICI therapy provides significant efficacy in cancer treatment, but it also carries a high risk of severe side effects. The correlating frequency of irAEs can impact several organs and complicate treatment (Figure 2). The current management strategies focus on comprehending the processes underlying these adverse effects and implementing solutions that utilize immunosuppressive drugs such as glucocorticoids (Ruf et al., 2024). Using a multidisciplinary approach to control irAEs is becoming a standard practice in the clinic. As the researchers aim to mitigate these adverse events, they are developing next-generation immune checkpoint inhibitors (ICIs) that target immunological checkpoints like TIGIT, TIM-3, and LAG-3. For patients not responding to conventional treatments, these innovative medicines may overcome resistance to existing medications and improve patients’ health.

## 4 Mechanisms of cancer resistance to immunotherapy

Despite providing better long-term efficacy as a first line treatment in NSCLC, Melanoma, Renal cell carcinoma and various advanced forms of cancers including bladder, head and neck tumors, ICIs show modest effect in overall population, due to majority of patients displaying primary and acquired resistance, alongside heterogenic response across various tumor legions in the same responding patient. Cancer resistance to immunotherapy can arise due to intrinsic and extrinsic factors. Intrinsic mechanisms comprise alterations of antitumor response pathways, changes in signaling pathways, and disruptive changes in tumor cells, which lead to an inhibitory, immunosuppressive microenvironment around the tumor. Variable tumor antigen concentration due to reduced/lost expression of antigens lowers immunogenicity, mediating tumor escape. Gene mutations abrupt signaling in antigen presentation signal pathways, drastically impacting transporters, MHC structure, and regulatory mechanisms, thus actively leading to resistance. Cytokines like Interferon-γ (IFN-γ) are produced by T cells and mediate upregulation of CXCL9, CXCL10, CXCR3+ lymphocytes, and other immune cells via JAK2 activation, leading to anti-tumor immune effects. IFN-γ can increase PD-L1 expression on tumor cells, thus enabling tumor escape. In recipients of immunotherapy, tumor cells may downregulate IFN-γ by loss-of-function alleles of genes that code for JKA1/2, STAT1, resulting in resistance to immunotherapy. Furthermore, tumor cells can secrete PD-L1 directly, mediating apoptosis in T cells and upregulating pro-tumorigenic Tregs (Nagasaki et al., 2022). Similarly, aberrations in cancer signaling pathways (MAPK/PI3K-AKT/WNT/β-Catenin) can affect sensitivity to immunotherapeutics.

Extrinsic factors comprise inadequate tumor infiltration by immune cells, compensatory upregulation of alternative checkpoint molecules, abnormal angiogenesis, and epithelial-mesenchymal transition (EMT). Tumor microenvironment (TME) has been broadly categorized into three categories, namely,: Infiltrated-inflamed (infiltrated by T cells in tumor parenchyma, thus leading to beneficial outcomes in ICIs), infiltrated excluded TME (surrounding tumor stroma infiltrated by immune cells), and immune desert TME (poor infiltration by immune cells). The latter two subtypes have been linked to resistance in immunotherapy. Pro-angiogenic agents like VEGF have shown suppression of cytotoxic CD8^+^ cells, while enhancing Tregs, myeloid-derived suppressor cells (MDSCs), and immune checkpoint molecule expression (Alsaafeen et al., 2025). EMT involves the transition of epithelial cells to mesenchymal cells, gaining properties of motility and invasiveness. EMT downregulates immune checkpoints in cancer cells, reducing susceptibility to PD-L1 therapy. It also further promotes recruitment of Tregs, IL-6, and IL-8, leading to immune suppressive and increased neovascularization (Said and Ibrahim, 2023). In addition to the above-mentioned factors, systemic host factors like diet, physical activity can affect ICI therapy. Although ICIs treatment has made great strides in treating different malignancies, the complexities of immune response and the dynamic and heterogeneous nature of tumors pose a strong challenge. These complex mechanisms can be targeted by adjuvant treatments to ICIs, namely, radiotherapy, chemotherapy, and adoptive cellular therapies such as CAR-T therapy.

## 5 Combinational therapies in cancer immunotherapy

### 5.1 Combination of radiation therapy with immunotherapy

**FIGURE 3:**
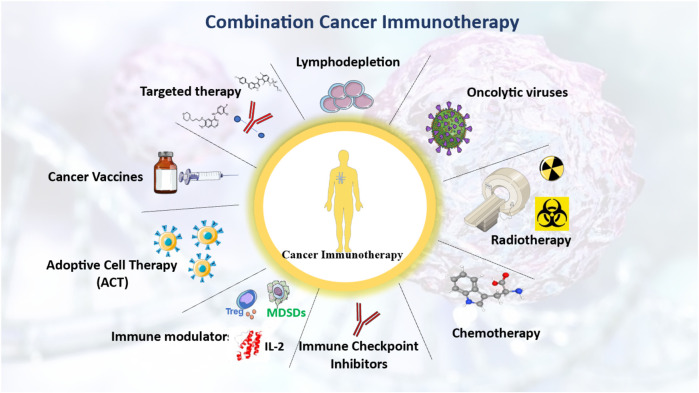
integrative strategies to enhance cancer immunotherapy through immune system modulation. Various treatment strategies that leverage the immune system to enhance cancer immunotherapy. Either stand-alone or as a combination of multiple approaches, such as chemotherapy, radiotherapy, and cancer vaccines, the overarching objective is to enhance the therapeutic outcomes of cancer immunotherapy. The figure contains modified images from Servier Medical Art, licensed under Creative Commons attribution 4.0 Unported License.

The first indications that radiation treatment (RT) can boost immunity against cancer originated from case studies in which inconceivable, untreated cancers diminished after local RT. With the development of ICI treatments, the fascinating potential of RT in eliciting an anti-cancer immune response has attracted a great deal of attention. Various approaches to cancer immunotherapy that can be combined to improve treatment outcomes have been reviewed (Figure 3).

### 5.2 Dual role of radiation in the modulation of anticancer immunity

Two essential elements of the immune response, i.e., enhancing both adjuvanticity and antigenicity were made possible by RT (Demaria et al., 2005). Through several mechanisms, RT can alter the local TME and increase antigenicity. RT, just like chemotherapy, promotes tumor antigen presentation by inducing MHC-1 expression (Liu et al., 2023). RT causes immunogenic cell death (ICD), in which APCs are guided to dying cancer cells by molecules such as Annexin A1, and antigen-uptake and presentation to T cells is promoted by HSP70, HSP90, and HMGB1 (Yamazaki et al., 2020). The release of HMGB1 exposes that radiation causes calreticulin (a protein that marks the cell for immune destruction) to translocate within the plasma membrane. RT increases the ability of APCs to recognize and absorb cancer cells by downregulating the “do not eat me” signal (CD47) on these cells (Yamazaki et al., 2020). Macromolecules are modified by reactive oxygen species (ROS) produced by RT, which increases their antigenicity. Also, these ROS can cause direct tissue destruction of cancer cells. RT increases adjuvanticity, which raises anti-cancer immunity even more.

RT has been shown to enhance anti-cancer immunity, but there is also strong evidence that it can induce an immunosuppressive TME (Demaria et al., 2015). In addition to killing cancer cells, radiation, especially broad-field radiation can harm normal immune cells. This can cause the TME to shift towards immunosuppression rather than increasing anti-cancer immunity. Additionally, radiation may raise the expression of activin A and TGF-β, which can draw in regulatory T cells (Tregs) and decrease the invasion of CD8^+^ T cells (Jarosz-Biej et al., 2019). Radiation also has additional immunosuppressive effects due to hypoxia, alterations in the stroma, damage of tumor blood vessels, and the influence of cytokines, TAMs, cancer-associated fibroblasts (CAFs), etc (Dancea et al., 2009).

### 5.3 Combination of chemotherapy and immunotherapy

Most chemotherapeutic drugs were created with the immune system’s impact contemplated rather than their immediate cytotoxic effects. Studies highlighting the combination of immunotherapy and chemotherapy have shown enhanced responses to anthracyclines in tumors, especially in mice models with intact immune systems, where notable outcomes have been obtained (Sordo-Bahamonde et al., 2023).

Numerous studies that have been conducted to date have demonstrated the potential of cytotoxic chemotherapy to enhance anti-cancer immunity. Harnessing anti-tumor immunity by adding one or more immunotherapeutic agents is one of the leading strategies currently being applied in clinical trials. Among the chemotherapeutics, alkylating agents (cyclophosphamide, carboplatin, cisplatin, and dacarbazine) and tubulin inhibitors are the major players that are being investigated in combination with Immunotherapeutics (Tsvetkova and Ivanova, 2022).

### 5.4 Combination of targeted therapy with immunotherapy

Targeting the genomic alterations harbored by oncogenes can induce more anti-tumor responses than cytotoxic chemotherapy. Among numerous ongoing clinical trials of combined immunotherapy and targeted therapies, the most efficient immunomodulatory responses are ascertained with anti-angiogenesis agents affecting almost all subpopulations of the immune system. Some of the FDA-approved combinations that target VEGF and other angiogenic proteins like platelet-derived growth factor receptor (PDGFR), c-kit, fibroblast growth factor receptors, etc., are Axitinib, Cabozantinib, Lenvatinib, and Bevacizumab (Ansari et al., 2022). The only combination therapy that doesn’t target angiogenesis directly is given in BRAF-mutated advanced melanoma, i.e., a combination of Vemurafenib (a BRAF inhibitor), cobimetinib (a mitogen-activated extracellular kinase (MEK) inhibitor), and atezolizumab (Morante et al., 2022). Other combinations like axitinib with pembrolizumab, axitinib with avelumab, and nivolumab with cabozantinib are prescribed in advanced renal cancer (Chen X. et al., 2024).

### 5.5 Combination of ACT with ICIs

PD1 upregulation after CAR-T cell infusion can optimize the co-stimulatory signaling of CD28, resulting in dysfunctional CAR-T cells. This suggests that CAR-T combined with PD1/PD-L1 blockade can achieve synergistic anti-tumor activity, as observed in many trials. Increased antitumor activity along with anti-PD-1 antibodies is noticed by modified CAR-T cells, i.e., PD1-deficient CAR-T cells, which cause no systemic toxicity. Along with PD1/PD-L1, other inhibitory immunosuppressive pathways have also been investigated with ACT, such as knockout of the TGF-β signaling, which is a major immunosuppressive regulator in CAR-T cells that increases CAR-T cell count, thereby showing efficient antitumor activity (Poorebrahim et al., 2021).

### 5.6 Combination of CAR-T cell therapy with lymphodepletion

Relapsed malignancies show no improvement with previously administered CAR-T cells, as patients might have developed an inhibitory immune response to them. To prevent such lymphodepletion, pre-CAR-T cell infusion has been employed that elevates APC activation by reducing the effect of Tregs and other immune cells, thereby facilitating increased CAR-T cells’ effectiveness (Davies and Maher, 2021).

### 5.7 Combination of two different CAR-T cell therapies

Combining two different structured CAR-T cells is an alternative strategy for zoning out the same target molecules instead of lymphodepletion. This can bypass the immune rejection developed against one type of CAR-T cells. Empirical evidence indicates that anti-CD19 CAR-T therapy (murine-derived) immune rejection can be overcome by humanized anti-CD19 CAR-T therapy (Nguyen et al., 2022). Another strategy to increase anti-tumor efficacy and avoid relapse is using CAR-T cells (containing a tandem build vector) targeting two different antigens on the same tumor cells.

### 5.8 Combination of CAR-T cells with immune modulators

Immunostimulatory molecules, such as cytokines and their receptors, and co-stimulatory molecules can be given in combination with the fourth generation of CAR-T cells. The fourth generation of CAR-T TRUCK cells expressing IL-12 is expected to treat solid tumors, which has not been possible with conventional CARs (Chmielewski and Abken, 2015), because IL-12 cytokines counteract the CAR-T suppression caused by Treg and MDSCs, and thereby improve the CD8 + T cells and NK cells cytotoxic activity, and stimulate the Th1 cell response against tumor cells.

### 5.9 Combination therapy with cancer vaccines

Cancer vaccines activate immunity against cancer via antigen presentation, priming, and activation of immune cells. Activated immune cells identify and eliminate cancer cells, mobilize and invade tumors, and elicit cytotoxicity. Dampened vaccine effectiveness can be caused by resistance mechanisms, particularly in the TME, for which researchers are still examining different treatment pathways. Priming T cells for anti-cancer immunity is the key intent of cancer vaccines (Fan et al., 2023), because many malignancies are not infiltrated by immune cells (“cold tumors”), and immune checkpoint blockers (ICBs) do not work on them (Rezaei et al., 2022). Vaccines are frequently used in conjunction with adjuvants like the TLR-3 agonist poly-ICLC to increase efficacy. For instance, when combined with poly-ICLC, the NY-ESO-1 vaccination exhibits an enhanced immune response (Zhou et al., 2023). Also, compared to IL-2 alone, the combination of IL-2 with gp100 (a tumor-associated antigen) showed significant improvement and progression-free survival (Choudhry et al., 2018). To increase the efficacy of cancer vaccines, researchers are investigating immunological co-inhibitory and co-stimulatory substances in addition to anti-PD1/PD-L1 and anti-CTLA-4 antibodies. A Dendritic cell MART-1 peptide vaccination and IMP321 (LAG-3Ig fusion protein) were administered together in a Phase I study. This combination showed potential for improving vaccination effectiveness as it enhanced MART-1-specific CD8 T cells and decreased the regulatory T cells (Zanotta et al., 2024).

## 6 Immunotherapy in hematologic malignancies vs. solid tumors

The fundamental principle of immunotherapy is the utilization of the immune system to target and kill malignant cells. Its efficacy has been proven in two broad categories of cancers: Hematologic cancers (such as leukemias, lymphomas, and myelomas), and solid tumors (such as lung, breast, and colorectal cancers). The principle remains unchanged across all the cancer types but the application, mode of treatment, and outcomes differ significantly between both hematologic cancers and solid tumors. Recent advances in cancer immunotherapy pave the path of new treatment strategies in hematological and solid malignancies to increase the benefits to the patient and minimize toxicity. Comparison of the efficacy, mechanisms, and challenges in treatment will shed light on differences in immunotherapy among these two types of malignancies (Pophali et al., 2024; Tang et al., 2023b).

Immunotherapy is largely successful in treating hematologic malignancies, especially for patients with relapsed or refractory disease. CAR-T cell therapy has achieved favorable outcomes and long-term remissions in specific hematological malignancies. Monoclonal antibodies, such as rituximab and daratumumab, provide significantly improved survival when combined with chemotherapy in B-cell lymphomas and multiple myeloma (Dong and Ghobrial, 2019). Trastuzumab is the first monoclonal antibody to be approved for a solid carcinoma, which targets the extracellular domain of the human epidermal growth factor receptor 2 (HER2/ErbB2) in breast cancer and other solid tumors (Cruz and Kayser, 2019). Immune checkpoint blocking therapy mainly consists of PD-1/PD-L1 inhibitors (nivolumab) and CTLA-4 inhibitors (ipilimumab). These inhibitors have shown positive therapeutic effects in the treatment of various malignant tumors (NSCLC and melanoma) (Wang et al., 2022). However, immunotherapy is also associated with side effects, including cytokine release syndrome (CRS) and neurotoxicity, and not all patients respond to the therapy. The efficacy of immunotherapy is more variable in solid tumors when compared to hematological cancers. Nonetheless, immune checkpoint inhibitors have changed the regime in melanoma and NSCLC. Unfortunately, other solid tumors (like colorectal, pancreatic, and ovarian cancers) show lower response rates. Primary and secondary resistance, as well as irAEs, are more common in solid tumors, which can restrict the effectiveness of immunotherapy. Immunotherapy is very beneficial in the treatment of cancer despite these problems, especially for treating hematologic malignancies.

TME is also important for immune therapy. Hematological malignancies majorly occur in the blood, bone marrow, and lymphatic system, which facilitates easy access to immune cells to recognize and interact with cancer cells. Solid tumors are surrounded by a complex microenvironment, including stromal cells, blood vessels, and an extracellular matrix that shields the tumor from immune attacks. Effective immunotherapy for treating solid tumors is severely hampered by the immunosuppressive environment, myeloid-derived suppressor cells, inhibitory cytokines, and Treg cells.

Leukemias, lymphomas, and other hematologic malignancies typically express a variety of antigens, including CD markers (CD19, CD20, etc.). Monoclonal antibodies or CAR-T cells in immunotherapy can successfully target these antigens. However, solid tumors often show more antigen heterogeneity, which makes it more challenging to find targets for immunotherapy that are universal or extremely specific (Tang et al., 2023b; Guzman et al., 2023).

## 7 Challenges in cancer immunotherapy

ICI treatment is challenged by the emergence of resistance mutations in biopsy samples obtained from melanoma patients, which have been linked to treatment duration affecting durable responses. These doubts can be addressed by carrying out further clinical trials and strategically employing artificial intelligence and machine learning, which extract the clinical and translational data to evaluate therapy-associated decisions (You et al., 2022). Pseudoprogression is characterized by an observed artificial increase in tumor size, while in fact, the anti-tumor effect has been exerted, leading to tumor shrinkage later on. As the therapies from tumor progression/regression inevitably vary, identification of key biomarkers such as circulating tumor DNA (ctDNA) concentrations, use of non-invasive biopsy for assessment of CD8^+^ and TIA1^+^ levels can aid therapy management (Ma et al., 2019). Hyperprogressive disease (HPD) is another phenomenon seen in ICI therapy, which is marked by accelerated tumor progression and progressive disease deterioration upon immunotherapy. T cells produce IFN-γ, which activates the β-Catenin pathway, mediating HPD symptoms in patients (Li et al., 2023).

CAR-T therapy targeting a single antigen may demonstrate loss of target antigen expression or tumor antigen heterogeneity, leading to relapsed or refractory myeloma. Use of dual/tandem CAR constructs reduces relapse rate, with CD19/CD22 CAR-T therapy displaying efficacy in adult patients with B-cell lymphoma (Klampatsa, 2024). Bispecific antibodies promote interactions between tumor cells and CAR-T cells, mediating cytotoxicity (Klampatsa, 2024). In contrast to hematological malignancies, solid tumors have high cell densities and lower vascular densities, leading to limited CAR-T cell infiltration and action. TME is immunosuppressive, due to the presence of tumor-associated macrophages (TAMs), Tregs, and MDSCs, that increase production of tumor-facilitating cytokines, chemokines, etc., whilst upregulation of immune checkpoint molecules reduces anti-tumor immunity. Under such circumstances, combinatorial treatment with ICIs, for example, PD-1 inhibitors and CAR-T therapeutics, was shown to enhance the therapeutic efficacy of CAR-T cells (Luo and Zhang, 2024). Additionally, setbacks such as severe toxicities including cytokine release syndrome (CRS) and the resultant immune effector cell-associated neurotoxicity syndrome (ICANS) pose challenges in approving CAR-T therapy as a first-line treatment. Cytokines are excessively produced and released by immune cells in CRS, leading to severe inflammation and results in tissue damage. Systemic inflammation resulting from CRS can impair blood-brain barrier function, leading to direct neurotoxic effects. Several agents have been tested for CRS and ICANS treatment, whilst IL-1 receptor antagonist Anakinra has shown some scope in treatment.

## 8 Future perspectives

Splicing Neo Antigen Finder (SNAF) is a computational research tool devised by an interdisciplinary research team from the University of Virginia and Cincinnati Children’s. It has the potential to identify shared immunotherapeutic targets that can be investigated further to design CAR-T cell therapies and other targeted treatment modalities. This also strengthens our understanding of tumor microenvironment. SNAF explores the repertoire of immunotherapy, discovering unbreached neoantigens occurring especially from splicing errors. For instance, SLC45A2, a prediction made by SNAF, turned out to be a highly reliable target due to its tumor specificity. The insights SNAF offers using the AI approach is making inroads into cancer immunotherapy (Li et al., 2024). CD99 tumor-associated antigen is found to be abundantly expressed on malignant T cells and thereby is a functional target for anti-CD99 mAbs aiding in the efficient treatment of T-cell acute lymphoblastic leukemia (T-ALL). V-domain Ig suppressor of T-cell activation (VISTA) is another upcoming target being studied for treating gastrointestinal tumors (Chmiel et al., 2023). The cyclic guanosine monophosphate-adenosine monophosphate synthase-stimulator of interferon genes (cGAS-STING) pathway internally regulates the immune system sensing of tumor growth. Considering this mechanism, STING agonists can be useful, but haven’t seen progress in clinical development due to poor pharmacokinetics (Motedayen Aval et al., 2020). Ubiquitin-specific proteases (USPs) are involved in tumor progression, and USP 7,14, and 22 inhibitors have been evaluated preclinically in this context. It has been observed that USP inhibition led to a remarkable increase in CD8 + T cells and natural killer cells, and transformed “cold” tumor into “hot” thereby sensitizing the tumor and making it more responsive to immunotherapy (Gao et al., 2023).

## 9 Conclusion

The design and development of a personalized treatment course that can uniquely target and block several immune checkpoints simultaneously can offer the advantage of preventing drug resistance. This improves the patient’s overall therapeutic outcomes. Spectacular evidence supporting the role of microbial consortia in promoting oncogenesis and modulating responses to cancer immunotherapy encourages designing treatment strategies that target and orchestrate the microbiome in a way that yields improved clinical outcomes. Thus, tuning the microbiota via probiotics or nanotechnology enables it to serve as a potential tool to prevent and treat cancer (Inamura, 2021; Cheng et al., 2020). A thorough understanding of immune-checkpoint regulation and the possibility of genetic manipulation of immune cells to magnify anti-tumor immune response needs to be reconnoitred (Kciuk et al., 2023; Mishra et al., 2022).
